# Attachment, Neurobiology, and Mentalizing along the Psychosis Continuum

**DOI:** 10.3389/fnhum.2016.00406

**Published:** 2016-08-22

**Authors:** Martin Debbané, George Salaminios, Patrick Luyten, Deborah Badoud, Marco Armando, Alessandra Solida Tozzi, Peter Fonagy, Benjamin K. Brent

**Affiliations:** ^1^Faculty of Psychology and Educational Sciences, University of GenevaGeneva, Switzerland; ^2^Research Department of Clinical, Educational and Health Psychology, University College LondonLondon, UK; ^3^Office Médico-PédagogiqueGeneva, Switzerland; ^4^Faculty of Psychology and Educational Sciences, University of LeuvenLeuven, Belgium; ^5^Department of Psychiatry, Lausanne University Hospital (CHUV)Lausanne, Switzerland; ^6^Department of Psychiatry, Massachusetts General Hospital, Harvard Medical SchoolBoston, MA, USA

**Keywords:** schizophrenia, mentalizing, HPA, schizotypy, UHR, theory of mind, self

## Abstract

In this review article, we outline the evidence linking attachment adversity to psychosis, from the premorbid stages of the disorder to its clinical forms. To better understand the neurobiological mechanisms through which insecure attachment may contribute to psychosis, we identify at least five neurobiological pathways linking attachment to risk for developing psychosis. Besides its well documented influence on the hypothalamic-pituary-adrenal (HPA) axis, insecure attachment may also contribute to neurodevelopmental risk through the dopaminergic and oxytonergic systems, as well as bear influence on neuroinflammation and oxidative stress responses. We further consider the neuroscientific and behavioral studies that underpin mentalization as a suite of processes potentially moderating the risk to transition to psychotic disorders. In particular, mentalization may help the individual compensate for endophenotypical impairments in the integration of sensory and metacognitive information. We propose a model where embodied mentalization would lie at the core of a protective, resilience response mitigating the adverse and potentially pathological influence of the neurodevelopmental cascade of risk for psychosis.

## Introduction

According to contemporary conceptualizations, psychosis is a neurodevelopmental disorder emerging during late adolescence and/or early adulthood and associated with the final stages of brain maturation. However, neuroscience research suggests that experiences of social adversity during early childhood, such as attachment-related trauma (Read et al., 2014), may independently contribute to alterations of neural development and brain dysmaturational processes during adolescence/early adulthood in those who go on to have psychosis (Brent et al., 2014b). Whilst basic neuroscience and novel brain imaging techniques have increasingly shed light on the neural correlates of psychosis vulnerability during the later stages of the psychosis risk period (Brent et al., 2013), there remains a great need to identify earlier (i.e., preadolescent and early adolescent) indicators of psychosis vulnerability. Importantly, the preponderance of youths at genetic or clinical high risk (CHR) for psychotic disorders will never transition to a first episode of psychosis (FEP), suggesting that illness onset may at least partly result from a breakdown of psychological mechanisms supporting resilience. The nature of such psychological mechanisms, potentially attenuating psychosis risk, remain incompletely understood.

Following the work of Brent et al. (2014a) and Brent and Fonagy (2014), we provide the theoretical basis to support further research regarding two inter-related, early putative protective factors: attachment security and mentalizing (a social cognitive capacity fostered by attachment security), which may together heighten resilience to developmental interpersonal stress and moderate the risk for psychosis onset. To frame our subsequent discussion, we begin with a consideration of the clinical phases associated with the emergence of psychosis.

**Figure 1:**
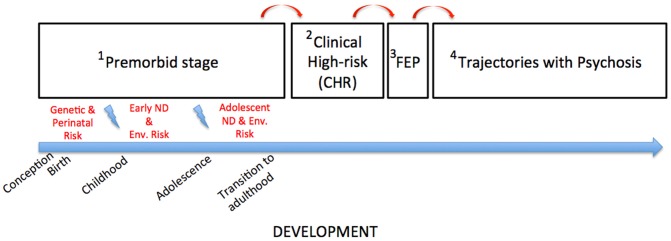
**The clinical developmental course of psychotic disorders, across the first two phases indicating increased vulnerability, and the last two phases charting the onset and development of the disorder.** FEP, First-Episode Psychosis; ND, Neurodevelopment; Env, Environmental.

The clinical course of psychotic disorders is commonly broken down into four phases (Figure 1): (1) the premorbid period, which is characterized by a wide-range of subtle physiological, neurocognitive and social impairments during preadolescent development that are thought to confer distal risk for schizophrenia (Seidman and Nordentoft, 2015); (2) the clinical high-risk (CHR), which encompasses a range of CHR states typically occurring during adolescence, or early adulthood (e.g., at-risk mental states (Yung et al., 2005), basic symptoms (Schultze-Lutter and Koch, 2010), ultra high-risk states (Miller et al., 2003), or significant functional decline in the context of genetic risk); (3) the first episode of psychosis (FEP), which marks the conversion to clinical psychosis; and (4) the post-conversion period, which can be divided into three developmental trajectories: remission, remission and relapse, and chronic disease.

Our specific interest concerns how the characteristics of the attachment environment may influence the trajectory of psychosis risk along its continuum of expression. We begin by highlighting the associations between trauma, insecure attachment, and psychosis. Second, we examine neurobiological links between early attachment trauma, stress, and social cognitive impairment. Lastly, we discuss the moderating role mentalization may play along the trajectory of risk for psychosis.

Our aim is not to construe a model of psychotic illnesses based solely on environmental risk factors (i.e., adverse attachment environment) acting in isolation, but rather to start fleshing out the kinds of transformations gene-environment interactions may incur during development, some of which appear to increase risk and probability to develop manifest psychotic disorders (van Os et al., 2010). We hope our review of the biological pathways upon which trauma, attachment and psychosis come together in gene-environment interactions and transactional processes may contribute to clarify which mechanisms may most likely lead to the emergence of psychotic disorders.

## Trauma, Attachment, and Psychosis

### Is There Evidence for an Association Between Trauma and Psychosis Along the Continuum of Psychotic Expression?

There is strong evidence suggesting the importance of trauma as a risk factor for psychotic disorders (Read et al., 2005; Varese et al., 2012). High rates of childhood trauma have been observed across the psychosis continuum, particularly when experiences of emotional and physical neglect are also included in the analyses. Recent retrospective studies have reported childhood trauma rates as high as 82% (Duhig et al., 2015) and 85% (Larsson et al., 2013) among samples of individuals with psychosis. In these studies, emotional abuse and neglect were found to be the most representative traumatic experiences, followed by physical neglect, sexual abuse and physical abuse. An important methodological issue of studies examining the incidences of childhood abuse in adult psychosis samples pertains to the retrospective nature of their assessment, which may be confounded by both normal processes of forgetting, but also by specific processes characterizing psychotic illnesses such as paranoid delusional ideation and cognitive impairment. A number of recent studies have addressed these limitations. For example, Fisher et al. (2011) examined the reliability of retrospective assessments of childhood trauma among patients suffering from psychosis, finding high levels of concurrent validity with measures of parental bonding and good convergent validity with clinical case notes. Most importantly perhaps, prospective longitudinal studies of children have shown that exposure to traumatic experiences early in life is predictive of the development of psychotic experiences (PE) over time with specific associations reported in relation to severity of trauma (Kelleher et al., 2013) and childhood abuse characterized by harmful intentions (Arseneault et al., 2011).

The harmful intentions abused children face often originate from caregivers (Sedlak et al., 2010). A number of different clinical accounts describe the affective double bind that abused children face. Briefly, a child facing a threat or danger automatically activates attachment needs that trigger approach mechanisms to the caregiver to ensure security. In cases where the caregiver is also a source of neglect and/or abuse, the child will have to manage the potential threat associated with proximity to the caregiver. The threat can be managed by adopting a hypervigilant stance towards the caregiver (as in avoidant-fearful attachment), which translates the child’s attempts to predict or control the threatening caregiver’s states of mind. Alternatively, or in combination, the child may also manifest avoidance behavior characteristic of avoidant attachment, in order to keep the source of threat at bay. Liotti and Gumley (2008) observe that it is not uncommon for children to dissociate in the face of abusive threats. In doing so the child’s attachment system is activated to maintain physical proximity and safety to the caregiver, whilst psychologically dissociating from the hostile images of the self that may populate the mind of the neglectful or abusing caregiver. This paradox lies at the core of the subjective experience of trauma; for some children, it can crystalize into disorganized attachment, which represents a combination of intense anxious and avoidant attachment strategies together with odd attachment behaviors which encapsulate the contradictory proximity-seeking and avoidant motivations of the child facing a maltreating caregiver (for example, walking backwards in the direction of the caregiver, to seek reassurance while avoiding visual and other straightforward contact; Fonagy et al., 2002).

Evidence suggests that traumatic experiences and sub-clinical psychotic manifestations are associated during childhood and adolescence. For example, Escher et al. (2002) found that 86.3% of a children sample reporting auditory verbal hallucinations (AVH; *n* = 80, mean age = 12.9 years) also reported experiencing traumatic and stressful life events in close temporal proximity to voice-hearing onset. Furthermore, prospective evidence links traumatic and stress-inducing events in childhood with the persistence and severity of auditory hallucinations, the emergence of new hallucinatory experiences in early adolescence, and the secondary development of delusional ideation (Bartels-Velthuis et al., 2012). Importantly, prospective studies in CHR populations have shown that childhood sexual trauma is predictive of conversion to a FEP, with individuals reporting high scores on sexual abuse questionnaires being 2–4 times more likely to transition into FEP compared to those with low scores (Bechdolf et al., 2010; Thompson et al., 2014).

Given the evidence linking adverse childhood experiences and the development of psychosis later in life (Read et al., 2014), and the association between early trauma and attachment insecurity, a number of authors have proposed that attachment security may be an environmental reliance factor, protecting against the likelihood that an underlying psychosis diathesis will become expressed (Read and Gumley, 2010). One possibility is that attachment security could foster protective psychological processes related to long-term adaptation, in the face of childhood traumatic events, while attachment insecurity may increase the risk of subsequent interpersonal dysfunction and psychopathological expression (Rutten et al., 2013).

### Attachment and Interpersonal Functioning Along the Psychosis Continuum

Initial studies by Dozier (1990) and Dozier et al. (1991) observed that psychiatric groups display significantly greater levels of attachment insecurity compared to healthy controls, with individuals suffering from schizophrenia showing higher levels of insecure attachment (particularly dismissing-avoidant attachment) than those suffering from affective illnesses. Consistent with these findings, high rates of insecure attachment have also been reported in more recent studies of patients with chronic psychotic disorders (Mickelson et al., 1997), and FEP (MaBeth et al., 2011). Additionally, meta-analyses have shown associations between adult attachment avoidance and both positive and negative symptomatology, with more modest evidence for an association between positive symptoms and attachment anxiety/preoccupation (Berry et al., 2007b; Korver-Nieberg et al., 2013; Gumley et al., 2014). Thus, both early attachment and adult attachment, which traditionally show some continuity over development (Waters et al., 2000), relate to symptomatic expression of individuals with psychotic disorders in retrospective and prospective studies.

Attachment security has also been associated with several important clinical outcomes, such as enhanced help-seeking and treatment engagement, as well as stronger therapeutic alliance among individuals with established schizophrenia (Berry et al., 2007b). By contrast, insecure attachment (attachment avoidance in particular) has been linked with reduced help-seeking behaviors, poor use of treatment (Dozier et al., 1991), diminished therapeutic alliance (Berry et al., 2008) and increased service disengagement (Tait et al., 2004).

Among psychosis prone individuals, the majority of studies looking at attachment has focused on schizotypal traits in familial or psychometrically defined risk samples. Data from non-clinical samples suggest that insecure avoidant attachment is associated with subclinical psychotic symptoms (Korver-Nieberg et al., 2013). Positive schizotypy has been associated with both attachment avoidance (Berry et al., 2007a; MacBeth et al., 2008) and attachment anxiety (Berry et al., 2007a), while negative schizotypal symptoms have been specifically linked to attachment avoidance (Berry et al., 2006, 2007a; Tiliopoulos and Goodall, 2009). In a large community study, preoccupied attachment was associated to positive schizotypy, dismissing attachment was related to negative schizotypy and fearful attachment was related to both positive and negative schizotypy (Sheinbaum et al., 2013). Given that preoccupied attachment is characterized by hyper-activation of the attachment system (Shaver and Mikulincer, 2002), these findings support the possibility that disruptions in the regulation of negative emotion, and increased salience of threat-related cues and distress may contribute to the expression and maintenance of positive schizotypy. On the other hand, dismissing attachment, characterized by a deactivation of the attachment system (i.e., interpersonal distancing, etc.), is significantly associated with negative schizotypy (Sheinbaum et al., 2013), which may contribute to persistent social withdrawal and isolation. In a recent study with a large sample of undergraduate students, fearful attachment was found to mediate the relationship between physical/emotional trauma and both positive and negative schizotypy (Sheinbaum et al., 2014). The authors suggest that individuals with fearful attachment hold negative working models of both self and others, and resort to antagonistic approach and avoidance strategies, which may contribute to emotional and cognitive disorganization (Sheinbaum et al., 2014).

Despite the developing interest in interpersonal and social cognitive functioning in high-risk for psychosis studies, only a limited number of investigations to date have examined attachment among CHR samples. In one cross-sectional study using the self-report Revised Adult Attachment Scale (Collins, 1996), 80% of CHR young adults showed evidence of insecure attachment (Gajwani et al., 2013). Further, in a prospective study with a sample of 31 ARMS individuals (mean age = 15.7 years), Quijada et al. (2012) examined the relationship between baseline attachment, symptoms and social functioning after a 6-month psychosocial intervention. The majority of individuals in this ARMS sample was classified, on the basis of the observer-rated Relationships Questionnaire (Bartholomew and Horowitz, 1991), as fearfully attached (71%), followed by preoccupied (16.1%) and dismissing attachment (6.4%). Improvement in attenuated positive symptoms at 6 months was associated with baseline secure, preoccupied and dismissing attachment styles. According to Quijada et al. (2012), secure, preoccupied and dismissing attachment styles, all share the presence of at least one positive internal working model indicating that psychotherapeutic interventions, at least during the early stages of psychotic illnesses, may benefit from a positive internal working model, either of the self or of others. Further, these data suggest that individuals who hold negative internal models of both self and others in the context of fearful attachment relationships may be at greater risk for a poorer clinical course. In a follow-up study, patients with lower levels of fearful and dismissing/avoidant styles at baseline, displayed better clinical outcomes (positive, negative and total symptom scores), after the implementation of a psychosocial intervention at 12 months follow-up (Quijada et al., 2015).

Overall, the available literature appears to support the view that: (1) attachment insecurity is associated with psychosis and psychosis-like symptoms throughout childhood and adolescent development; (2) attachment classification is linked with treatment engagement and other clinical outcomes. Here, positive internal working models of self and/or other speak to a favorable disposition to respond to psychosocial treatment. At the neurobiological level of analysis, the impact of attachment on the potential routes sustaining a disposition to develop psychosis still require to be further characterized.

## Neurobiological Trajectories Linking Attachment to Psychosis Vulnerability

It has been proposed that psychotic disorders result from early (perinatal) and later (adolescence) biological insults that engender pathogenic processes altering the course of normal brain maturation (e.g., dendritic pruning) in late adolescence and young adulthood (Keshavan, 1999; Fatemi and Folsom, 2009). In the previous section we reviewed evidence suggesting that early trauma, together with insecure attachment, may affect the symptomatic course, therapeutic outcome, and interpersonal functioning associated with psychosis. This is not to say that insecure attachment is inherently maladaptive; as suggested above, insecure attachment in the context of trauma is a likely adaptive reaction (Chisholm, 1996; Ellis et al., 2011) to regulate distance and proximity to unpredictable and potentially harmful attachment figures. However, as suggested by studies on therapeutic outcomes, insecure attachment may hinder the development of alternative secure bonds to other individuals, thereby neutralizing potential psychosocial protective factors along the progression of the illness. As we will further develop in the third section, we also posit that attachment plays a critical role in the development of mentalizing, which may constitute a key protective social cognitive mechanism in psychosis-prone individuals.

In this section we will specifically focus on the links between the neurobiology of psychosis and attachment-related stress, which will highlight how the development of social cognition may be undermined in the course of childhood and adolescent development. We will survey five biological markers of psychosis (hypothalamic-pituitary-adrenal-axis (HPA-axis) hyperactivity, dopamine dysfunction, reduced oxytocin, neuroinflamation and oxidative stress) and how they may interact with developmental adversity to influence both childhood and adolescent neurodevelopmental disturbances associated with the illness. Although severe childhood abuse and emotional neglect have also been associated with non-schizophrenic illnesses such as borderline personality disorder (BPD) (Fonagy and Luyten, 2009, 2016), we highlight that in the developmental unfolding of psychosis, the pathogenic neurobiological impact of early attachment adversity, particularly during critical periods of neurodevelopment (ND), may more severely affect brain regions sustaining the capacity to regulate self-generated experiences (body-states, thoughts, feelings). More specifically, echoing formulations from phenomenological psychiatry (Nelson et al., 2009; Sass, 2014), we suggest that social cognitive impairments in relation to self-generated bodily, affective and cognitive states along development, severely undermine the establishment of a coherent sense of self and promote a breakdown of self and reality monitoring.

### HPA-Axis Hyperactivity and Psychosis

Early-life adversity has been found to significantly affect later life stress-responses by inducing long-term dysregulation of HPA-axis function, particularly in terms of elevated diurnal cortisol levels (Heim et al., 2008). Similarly to individuals suffering from depression and/or post-traumatic stress disorder, increases in glucocorticoid levels (cortisol in particular) have been reported in samples of patients suffering from schizophrenia compared to healthy controls, suggesting a potential relationship between early-life stress and psychosis (Ryan et al., 2004; Mondelli et al., 2010). Indeed, among patients with schizophrenia, those with a history of childhood abuse have been found to display significantly greater HPA-axis hyperactivity compared to non-abused controls (Braehler et al., 2005). Further, a more recent longitudinal study reported increased cortisol levels among CHR individuals who transitioned to a FEP, consistent with research on chronic samples (Walker et al., 2010).

At the neurobiological level, stress-based glucocorticoid exposure during critical periods of ND significantly affects the structure and function of brain regions with a high density of glucocorticoid receptors (e.g., hippocampus and prefrontal cortex) as well as regions sensitive to repeated neuronal excitation (e.g., amygdala; Teicher et al., 2003; McCrory et al., 2011). Functional and structural changes in these areas have an important influence on the disruption of cognitive processes associated with psychosis. For example, Aas et al. (2012) reported that childhood trauma was associated with smaller amygdala volume in a group of FEP subjects. Reduced amygdala volume also mediated the relationship between childhood trauma and impaired executive functioning. In addition, the disruptive effects of cortisol on prefrontal cortical activity have been linked with impaired higher order social cognitive processes (Arnsten, 2009). Specifically, acute stress has been shown to affect the dorsolateral and medial prefrontal cortex (mPFC), leading to a transient switch from flexible and reflective processing of social information, to a more automatic, action-based one (Fonagy and Luyten, 2009; Reyes et al., 2015). Additionally, chronic exposure to stress can produce extensive structural alterations in the PFC, including the loss of dendritic length, branching and spine density (Arnsten, 2009). Functional neuroimaging data from healthy participants suggest that the ventral and dorsal mPFC constitutes part of a wider network of cortical midline structures associated with self-referential and self-other discriminative processing (Brent et al., 2014b). Chronically elevated corticosterone levels in the context of adverse caregiving environments therefore may hinder the integrity of brain regions underpinning self-referential processing, leading to premorbid difficulties, in the capacity to differentiate between self and non-self cues, during middle childhood (Brent et al., 2014b). In line with two-hit neurodevelopmental conceptualizations of psychosis (Keshavan, 1999), adolescence-specific interpersonal stressors may thus further compromise the already vulnerable prefrontal self-processing network, leading to increased difficulties in self-reflective and self-monitoring processes as well as in other social-cognitive domains, and eventually to prodromal psychotic manifestations (Brent et al., 2014b). In this vein, psychosis would not directly emanate from HPA dysfunction; rather, the HPA dysfunction sustained by trauma and insecure attachment, would contribute in bringing the individual closer to the clinical threshold of psychotic breakdown. In contrast to exposure to trauma and neglect, supportive early social experiences associated with maternal care appear to promote the modulation HPA-axis-based threat reactivity, thus fostering flexibility and resilience in the face of novel stress-inducing social situations (Gunnar and Quevedo, 2007). By undermining the neural integrity of brain regions sustaining flexible and robust emotion regulation, as well as self-referential and metacognitive processing, the early disruption of the stress regulatory system may, therefore, make an important contribution to psychosis risk.

### Dopamine Dysfunction, Oxytocin and Psychosis

For the past 40 years, the role of dopamine in the development and maintenance of psychosis-spectrum illnesses has been supported by a large number of biological, neuroimaging and genetic studies (Carlsson, 1977; Howes and Kapur, 2009). Initial evidence linking the effectiveness of antipsychotic medication to changes in dopamine receptors was followed by studies utilizing advances in neurochemical imaging to directly examine dopamine levels among patients suffering from psychosis. These studies consistently report increased presynaptic striatal dopamine levels in psychotic illnesses, particularly during acute phases (Howes and Kapur, 2009). Furthermore, 4 of the 10 candidate genes associated with schizophrenia are directly linked with dopaminergic pathways (Howes et al., 2004; Howes and Kapur, 2009). Advances in research methodologies have led to further investigations in the effects of dopaminergic abnormalities on clinical phenotypic manifestations in psychosis.

Robust evidence indicates that mesolimbic dopamine guides motivational responses by assigning neural significance to external stimuli. Through this process, neutral external information is transformed into engaging or aversive entities on the basis of previous experience and predisposition. It has been consistently shown that in psychosis, dysregulation in dopamine transmission results in stimulus-independent release of dopamine (Kapur, 2003; Kapur et al., 2005). This neurochemical aberration disrupts normal processes of stimuli-based salience, leading to states of heightened awareness (“aberrant salience”) with respect to both external and internal stimuli (Kapur, 2003; Howes and Kapur, 2009). According to Kapur (2003), positive psychotic symptoms, particularly delusions and hallucinations, develop over time as personal explanations of the distressing experience associated with aberrant salience.

Recent studies have linked elevated striatal dopamine among CHR individuals, with greater severity of positive symptoms (Howes et al., 2009). Increased dopamine levels in the striatum have also been reported among schizotypal individuals (Soliman et al., 2008) and first-degree relatives of patients with schizophrenia (Huttunen et al., 2008), suggesting that dopaminergic abnormalities could underpin psychosis proneness (Howes and Kapur, 2009).

Pertinently, stress among at-risk individuals has been associated with both psychotic symptom severity and dopamine levels (van Winkel et al., 2008). Data suggest a synergistic relationship between the HPA-axis and the dopaminergic system (Phillips et al., 2006). More specifically, evidence from animal studies indicate that early life adversity within the caregiving environment is linked with chronic dysfunction of the dopaminergic system, particularly in terms of increased rates of dopamine synthesis and release in response to acute stress (Strathearn, 2011). Furthermore, chronic exposure to stress has been shown to decrease tonic activity in the midbrain, normally responsible for regulating dopamine levels in the nucleus accumbens (Phillips et al., 2006). According to neurodevelopmental accounts of psychosis (Keshavan, 1999), dopaminergic disregulation during the premorbid phase of the illness is modulated by a relatively intact PFC, which exerts inhibitory control over subcortical striatal dopamine. In adolescence, however, the derailment of normal maturational processes (i.e., synaptic pruning), as well as a normative increase in basal firing (Luciana et al., 2012), may lead to stress regulatory demands exceeding prefrontal function, and subsequently to the depletion of PFC-based subcortical dopaminergic modulation (Keshavan, 1999).

A complementary account argues that attachment insecurity may worsen dopaminergic dysfunction because of decreased levels of available oxytocin (Brent et al., 2014a). Oxytocin is critical for the regulation of early infant-caregiver relationships (Feldman et al., 2010) and the establishment of later social-affiliative behaviors (Wismer Fries et al., 2005). Oxytocin neurones project to brain structures responsible for the stimulation of maternal behaviors (Strathearn, 2011), and oxytocin facilitates physical proximity and care within mother-infant interactions (Insel, 2010). Evidence from rodent studies suggests that oxytocin administration stimulates a range of maternal behaviors while administration with an oxytocin antagonist inhibits maternal care and induces neglectful maternal attitudes (Pedersen et al., 1994). The prosocial effects of oxytocin have been specifically attributed to its stress regulating properties (Tas et al., 2014). According to Taylor et al. (2006), within stressful interpersonal interactions, hypothalamic oxytocin release modulates stress and rejection-related ideas, allowing individuals to constructively relate to others. Indeed, a number of non-clinical studies have reported that central oxytocin administration improves social cognition and social behavior (Tas et al., 2014). Importantly, although oxytocin facilitates the onset and maintenance of maternal caregiving behavior, the quality of the caregiving environment itself, stimulates the development of the oxytocinergic system in the offspring (Strathearn, 2011). Animal and human studies suggest that non-maternal rearing is linked with decreased levels of cerebrospinal fluid oxytocin during the first years of life (Winslow et al., 2003; Heim et al., 2009) and reports of childhood emotional neglect are negatively correlated with cerebrospinal fluid concentrations (Heim et al., 2009). Thus, adverse experiences within early attachment relationships may exert a prolonged impact on the development and function of the oxytocinergic system.

In schizophrenia, studies have shown associations between reduced serum levels of oxytocin and difficulties in facial emotion identification (Goldman et al., 2008), reduced plasma levels of oxytocin among patients during trust-dependent interactions with others (Kéri et al., 2009), as well as negative associations between oxytocin levels and severity of psychotic psychopathology (Rubin et al., 2010). According to Tas et al. (2014), the oxytocinergic system affects social cognition by acting upon subcortical structures (i.e., amygdala) responsible for basic social cognitive processes (i.e., facial emotion recognition), which in turn affect cortical areas (i.e., PFC), responsible for higher order metacognitive processes such as theory of mind (ToM). While interpersonal arousal in the context of insecure attachment may lead to transient disruptions in cortical brain areas responsible for metacognitive processing (i.e., mPFC, posterior cingulate cortex (PCC)), in healthy subjects the oxytocinergic system modulates amygdala-based stress and promotes mutually beneficial contingent relational responses (Bakermans-Kranenburg and Van IJzendoorn, 2013). The prosocial effects of oxytocin appear to be particularly pertinent through their modulation of avoidant attachment responses.

Data from randomized control trial (RCT) studies in healthy participants suggest that intranasal oxytocin administration enhances the experience of attachment security (Buchheim et al., 2009) and leads to increases in trust and cooperation along with reductions in betrayal aversion (De Dreu, 2012), specifically among those classified as avoidant attached at baseline. The impact of the oxytocinergic system to psychotic psychopathology may thus critically depend on attachment dimensions, and in particular the degree of avoidant attachment displayed by individuals prone to or suffering from a psychotic disorder. In a similar fashion, reduced oxytocin levels may sustain the premorbid social functioning patterns (distrust, social withdrawal and isolation) displayed by children and adolescents who go on to develop psychotic disorders, significantly depriving them of mutually beneficial interpersonal interactions that promote the development of social cognition (Fonagy and Luyten, 2009; Vrtička et al., 2014). Indeed, while trust related interactions are shown to be associated with increased oxytocin levels in healthy controls, the same pattern is absent in patients suffering from schizophrenia (Kéri et al., 2009). In the context of a genetic diathesis for psychosis, attachment insecurity and concomitant depletions of available oxytocin could, therefore, amplify the vulnerability to dopamine dysregulation and heighten the vulnerability to aberrant salience and low-grade psychotic symptoms. It is important to note however that more studies are needed to inform our understanding of the relationship between oxytocin levels and psychotic symptomatology, as well as on the effects of oxytocin on the development of trust in clinical samples. For example, current evidence on the therapeutic effects of oxytocin administration in the treatment of psychotic symptoms are mixed (Feifel et al., 2016; Lee et al., 2016) and although oxytocin administration is shown to facilitate trust and corporation in healthy samples, the opposite effect has been observed in some studies involving patients suffering from BPD (Bartz et al., 2011; Ebert et al., 2013).

### Neuroinflammation, Oxidative Stress and Psychosis

Inflammation refers to one of the organism’s first lines of defense against pathogenic infections or injuries and chronic inflammation has been associated with the pathophysiology of various physical and psychiatric illnesses (Kirkpatrick and Miller, 2013). Chronic inflammation in the brain (neuroinflammation), leading to increased microglial activation and inflammatory cytokine release, can disrupt the blood-brain barrier, which normally controls the entry of cytokines and other substances in the brain, causing alterations in brain function (Kirkpatrick and Miller, 2013). A number of studies have reported that individuals suffering from schizophrenia display elevated blood concentrations of inflammatory cytokines compared to healthy controls (Miller et al., 2011). Moreover, blood concentrations of certain inflammatory molecules in psychosis show variations according to clinical status (i.e., higher cytokine blood concentrations than controls during the exacerbation of the illness and no significant differences during periods of clinical remission; Miller et al., 2011). Importantly, neuroinflammatory abnormalities have been found among both FEP subjects and antipsychotic-naïve relatives of patients with schizophrenia, thus independent of antipsychotic medication use (Martínez-Gras et al., 2012). Among patients suffering from schizophrenia, increased blood cytokine levels have been associated with worse cognitive functioning along with measures of regional brain volume and negative psychotic pathology (Kirkpatrick and Miller, 2013). Perkins et al. (2015) reported increased plasma concentration of 15 proinflammatory analytes among CHR subjects that progressed to formal psychosis compared with non-converting CHR subjects and healthy controls, suggesting that inflammation may be a prominent factor in the early stages of psychotic illnesses.

Animal studies suggest that inflammation during critical periods of ND may significantly impact on the “set-point” of the inflammatory system, affecting subsequent inflammatory responses displayed in adulthood (Bilbo and Schwarz, 2009). Indeed, inflammation may mediate various recognized prenatal and perinatal risk factors for schizophrenia such as preterm labor, maternal gestational diabetes, preeclampsia, but also maternal depression and anxiety often associated with insecure attachment (Kirkpatrick and Miller, 2013). For example, (Buka et al., 2001) reported that maternal serum concentrations of specific cytokines (IL-8 and TNF-alpha) during pregnancy were associated with an increased risk of schizophrenia in the offspring.

According to O’Connor et al. (2014) cortisol and other glucocorticoids have clearly demonstrated effects on the immune system, suggesting a bidirectional link between stress and the immune system. Decreased receptor sensitivity to glucocorticoids and increased glucocorticoid exposure resulting from prolonged stress may reduce the anti-inflammatory effect of glucocorticoids, leading to persistent inflammation experienced by stressed individuals and subsequently to the development of various physical and psychological illnesses (O’Connor et al., 2014).

Further, Cannon et al. (2015) longitudinally investigated gray matter changes and plasma based markers of inflammation in a large CHR sample. MRI data indicated that CHR individuals who went on to develop formal psychotic psychopathology showed a steeper rate of gray matter loss in frontal areas and a greater rate of expansion of the third ventricle compared to non-converters and healthy controls. Higher levels of plasma based proinflammatory cytokines at baseline were strongly predictive of the observed steeper rates of gray matter reduction in right PFC areas among CHR converters. Bloomfield et al. (2016) used PET imaging to compare microglial activation between individuals at CHR for psychosis and healthy controls. Data revealed that microglial activation was significantly increased in total, frontal and temporal lobe gray matter in the CHR group compared to healthy controls.

As mentioned above, the PFC modulates the response of subcortical regions to stress and provides inhibitory feedback on the HPA-axis (Teicher et al., 2003). Furthermore, prefrontal regions are essential for the development of multiple processes of social cognition (Lieberman, 2007). It is possible that cortical thickness loss around the onset of psychosis significantly alters the capacity to regulate certain stressors in individuals at CHR, stressors that will inevitably come in the interpersonal domain during adolescence (most notably interpersonal stress, such as bullying; Mackie et al., 2013; Trotta et al., 2013). Additionally, the evidence for increased microglial activation in the frontal and temporal lobes of CHR individuals (Bloomfield et al., 2016) as well as the observed steeper rates of prefrontal gray matter loss, associated with proinflammatory cytokine levels, close to psychosis onset (Cannon et al., 2015), suggest that through their pathogenic effects on prefrontal brain maturation, neuroinflammatory processes may contribute to the alteration of neurodevelopmental mechanisms underpinning self-referential and socio-cognitive processing in adolescence. Along these lines, a recent study by Piskulic et al. (2016) charted the development of different social cognitive domains in a group of 764 CHR participants. Whilst no differences were observed in baseline social cognition between converters and non-converters, the latter were the only group to show significant developmental gain in social cognitive domains over a 1-year interval (Piskulic et al., 2016). The continued improvement in social cognition in non-converters should be further charted along development, as it could critically enable at-risk individuals to regulate the arousal evoked in the context of interpersonal relationships. On the other hand, the levelling-off of social cognitive development around the conversion period to psychosis in converters could mark the attenuation of protective factors opening the gate to psychopathological processes and resulting in severe morbidity in the social domain as observed in chronic schizophrenia.

In addition to inflammation, emerging evidence suggest that the pathophysiology of schizophrenia may be, at least in part, a consequence of oxidative stress due to aberrant reduction-oxidation (redox) control (Bitanihirwe and Woo, 2011). Data from human and animal studies indicate that adverse interpersonal experiences play an important role in sustaining the links between oxidative stress and psychosis (Möller et al., 2011; Aydin et al., 2015). More specifically, according to Do et al. (2009), in psychotic illnesses, genetically based disruptions in antioxidant control (i.e., reductions in glutathione (GSH)) interact with pro-oxidative environmental risk factors (including childhood and adolescent attachment adversity and stress) during critical periods of ND, to significantly disrupt the processes of neural connectivity and synchronization, particularly in relation to prefrontal areas associated with self-referential and socio-cognitive processing (Brent et al., 2014b).

Clinical and preclinical investigations into the mechanisms of antioxidant defenses in the brain suggest multiple pathways through which chronic oxidative stress can affect the development and course of psychotic disorders. Specifically, multiple studies have reported alterations in antioxidant enzymes in schizophrenia (Bitanihirwe and Woo, 2011). Data from a meta-analytic study indicated an increase in the level of lipid peroxidation products and nitric oxide (NO) in schizophrenia along with significantly decreased activity of antioxidant enzymes (Zhang et al., 2010). Moreover, levels of plasma antioxidants are significantly reduced in the illness, independently of other prooxidative factors such as smoking (Yao et al., 2000). Clinical trials provide further support to the association between oxidative stress and schizophrenia. RCT data showed that treatment with the antioxidant N-acetylcysteine significantly reduced psychotic pathological manifestations (Berk et al., 2013). Furthermore, Amminger et al. (2010) found that supplementation with fish oil reduces the progression to FEP in UHR subjects, with baseline levels of specific omega-3 polyunsaturated fatty acids predicting treatment response (Amminger et al., 2015). Additionally, evidence from preclinical studies with rodents show that alterations in antioxidant systems may underlie cognitive impairments and biochemical changes that are relevant to schizophrenia (Cabungcal et al., 2007).

Animal models provide information into the potential mechanisms linking psychological stress to the pathogenic effects of oxidative stress in the brain. In one rodent study, Wilson et al. (2013) reported that in comparison to control rats, rats in a stress induction condition exhibited slower growth, higher plasma corticosterone levels, greater anxiety-like behavior and a dose response relationship between duration of stress exposure and levels of reactive oxygen species (ROS). Post mortem analysis of brain tissue showed increased levels of oxidative stress parameters in the hippocampus and PFC in the stress-manipulated rats (Wilson et al., 2013). According to Miller and Sadeh (2014), animal models suggest that HPA-axis-based glucocorticoid release under stress is associated with increased ROS and oxidative damage. Additionally, longer duration of glucocorticoid administration has been linked with greater oxidative damage (Costantini et al., 2011) and oxidative stress has been shown to have a mediating role in the effects of glucocorticoids on neurodegeneration (Sato et al., 2010).

Given that early life trauma is a known risk factor for schizophrenia, Möller et al. (2011) examined the association between social isolation rearing (SIR) and oxidative stress in rats. SIR rats displayed profound deficits in social interaction as well as in sensory-motor gating (basic inhibitory process of regulation of sensory input preventing the cognitive fragmentation and sensory overload typical of schizophrenia). SIR led to significantly elevated levels of lipid peroxidation in both the frontal cortex and the striatum of SIR rats, suggesting that both SIR-induced sensory-motor and behavioral changes were associated with increased cortico-striatal oxidative stress (Möller et al., 2011).

In human studies, Aydin et al. (2015) examined the impact of caregiver attachment style and expressed emotion on schizophrenic participant’s oxidative stress parameters. Results showed that reduced and oxidized forms of glutathione (GSH and Glutathione disulfide, GSSG), plasma lipid peroxidation and urine malondialdehyde (MDA) levels of patients were higher compared to those reported in the healthy control group. Pertinently, regression analysis revealed that the main significant predictors of patients’ GSSG oxidative stress level were their caregivers’ emotional over-involvement and anxious-ambivalent attachment style. These data suggest that factors related to interpersonal stress within caregiving relationships may have a significant effect on oxidative stress parameters in psychotic illnesses.

The findings from human and animal studies reviewed above, suggest that traumatic early life stress may promote oxidative damage in the brain, which is shown to both alter processes of ND (Do et al., 2009) and enhance processes of neurodegeneration (Sato et al., 2010). In a pioneering study, Do et al. (2000) reported that GSH levels, which play an important role in protecting against oxidative damage, were reduced by 52% in the mPFC of drug naive patients with psychosis compared to a healthy control group. According to these authors, a deficit in GSH during early development constitutes an important risk factor for psychosis development, as it promotes neurodegenerative processes that lead to a loss of neural connectivity in various brain regions, including the PFC. This observation may relate to Brent et al. (2014b), who underline resting state fMRI studies where greater alterations of mPFC functional connectivity among individuals at genetic high-risk is associated with greater levels of psychotic manifestations. Here, we simply wish to underline that sustained oxidative stress, particularly during early ND, may heighten the risk for psychotic disorders later in life, causing structural and functional brain changes in regions associated with self-processing, a wide-range of cognitive processes (such as spatial learning, spatial memory (Cabungcal et al., 2007)) and sensory-motor gating (Möller et al., 2011). Interestingly, NMDA receptors as well as GABAergic interneurons are also thought to sustain the critical relationship between sensory and metacognitive signaling (Adams et al., 2013), which we will further discuss in the final section.

**Figure 2:**
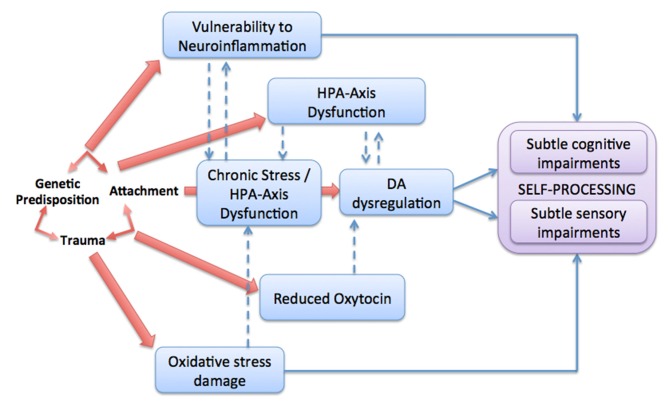
**Illustration of the five neurobiological pathways (red arrows) through which attachment adversity (both during childhood and adolescence) may augment risk for psychosis.** Dotted arrows represent potential interactions between pathways.

Overall, our review of the literature highlights that the aberrant neurobiological processes underpinning psychosis proneness and development: (1) are influenced by early adverse, stress-inducing experiences in the context of caregiving attachment relationships, particularly during critical periods of ND; (2) they all, at least in part, exert their pathogenic impact by making the self-processing fronto-tempo-parietal system more vulnerable; and (3) They are associated with sensory and cognitive impairments early, which can critically impact self-processing (Figure 2).

## Mentalizing as A Moderator of Psychosis Vulnerability

In the first section of this article, we reviewed the different strands of evidence underlining the relevance of attachment relationships at each phase of developing psychosis. On the one hand, early attachment insecurity is overrepresented along the continuum of expression of psychosis, from the premorbid manifestations, through the CHR states and into the clinical range of psychosis. On the other hand, attachment security appears to be linked to an increased capacity to engage in psychosocial treatment and to benefit from these interventions, at least in adults diagnosed with a psychotic disorder. In the second section, we presented five neurobiological pathways that fundamental research in neuroscience has revealed to represent possible routes taken by pathogenic processes towards the emergence of psychotic disorders. Importantly, each of these pathways may individually contribute to undermine the regulation of self-experiences, from basic sensory-gating processes to inferential processes about self-generated contents, including bodily experiences, feelings and thoughts; most importantly perhaps, they undermine the very processes that scaffold the illusion of a coherent and stable self, namely mentalizing or social cognition. Indeed, these neurobiological pathways may shed some light into the mechanisms underlying disturbed self-experiences (Brent et al., 2014a,b).

In this third and final section, we will explore the hypothesis that the suite of processes falling under the umbrella term of *mentalizing* may act as moderators of risk for transitioning to psychosis. We will build our case on evidence suggesting that early impairments in mentalizing, caused either by environmental factors (e.g., trauma), biological factors or a combination of both, are associated with sub-clinical manifestations of psychosis at the trait and state levels of expression. Second, we review evidence suggesting that robust mentalizing during adolescence is associated with the natural resolution and the disappearance of hallucination and delusion-like phenomena. Finally, we propose a bridge with the computational psychiatry account of psychosis to reduce the divide between social cognitive state manifestations of psychosis and sensory-based, trait markers of risk for psychosis. We argue that processes falling under the rubric of *embodied mentalizing*, the capacity to experience the body as the seat of emotional responses and to reflect on the relationship between bodily states and mental representations (Luyten et al., 2012) may enhance our understanding of the potential aberrations in the relationship between sensory-affective experience and metacognitive certainty.

### Evidence of Early Impairments in Mentalizing Along the Psychosis Continuum

Mentalizing is a multidimensional construct that signifies attempts to understand oneself and others as driven by intentional mental states, such as beliefs, desires, emotions or intentions (Fonagy and Luyten, 2009; Bateman and Fonagy, 2012). A number of phenomena related to psychosis entail aberrant mentalizing. At the most concrete level, individuals with psychosis may develop rigid convictions about others’ intentions based solely on their physical observable behavior, while losing the capacity to consider alternative perspectives on the basis of internal mental states (teleological mentalizing; Fonagy and Bateman, 2006), such as in cases where others’ behavior gives rise to paranoid delusions. For example, a patient describing random eye contact with a stranger on the bus may say “*He turned around and looked at me, clearly wanting to hurt me*”. They may also experience their thoughts about themselves or others as isomorphic to reality (psychic equivalence; Fonagy and Target, 1996). Ideas of reference constitute a good example of mentalizing in psychic equivalence, because the individual denies the opacity of others’ states of mind (i.e., an idea of reference held in psychic equivalence: “*I feel that the news broadcaster is hinting a message specifically addressed to me*”). Finally, phenomena such as hyper-reflexivity characterize a type of mentalizing activity that has dissociated from present perceptual, sensory and affective content (mentalizing in pretend mode; Fonagy and Target, 1996). For example, psychotic patients in psychotherapy often develop a hyper-reflexive mode of discourse characterized by intricate and convoluted explanations of self and others’ mental states, a discourse that will typically be detached from the patient’s current emotional arousal (for example, immediate feelings vis-à-vis the relationship to therapist). In such instances, the therapist will often experience feeling lost or confused by the patient’s discourse held in pretend mode mentalizing (Debbané et al., 2016).

Developmentally, mentalization is fostered by secure attachment. A number of studies have shown that secure attachment is positively associated with the normative development of emotion recognition, ToM, and mind-mindedness (Fonagy et al., 2002). The caregiver’s contingent and *marked mirroring* (attuned yet slightly distorted reflection) of the infant’s emotional communication is hypothesized to provide a mentalizing playground where second-order representations about self-experience can be elaborated in safe and playful interactions (Gergely and Watson, 1996). Later on, mature mentalization builds on the linking of specialized social cognitive functions, which necessarily interact in the adult’s mind to construe accurate interpretations of complex social behaviors as motivated by underlying intentional mental states of self and others. In essence, the articulation and binding of processes giving rise to mentalization represent a developmental achievement that critically depends on interacting systems, or in neuroscientific terms, on multi-layered connectivity within and between systems that sustain self and other monitoring, and more broadly, social cognitive functions. It may thus not appear entirely surprising that psychotic symptoms, most consistently characterized as coming from “dysconnectivity” (Friston, 1999; McGlashan and Hoffman, 2000), present themselves in forms of aberrant mentalizing of self and others.

Previously, Brent and Fonagy (2014) have proposed that among genetically predisposed individuals, mentalizing deficits particularly in the context of stress-inducing experiences associated with early attachment disturbances, may interact with dopaminergic dysfunction to facilitate the breakdown of reality testing and the development of psychosis. To date however, mentalizing deficits in psychosis have been primarily investigated through the use of ToM tasks, which overlap only with the cognitive, other-oriented portion of mentalization. Meta-analytic data from ToM studies have confirmed the presence of stable mentalizing deficits in the course of psychotic illnesses, independent of IQ, age and gender (Harrington et al., 2005; Sprong et al., 2007). Moreover, ToM disturbances have been associated with positive psychotic symptoms (paranoid delusions in particular; Harrington et al., 2005), poor illness insight (Bora et al., 2007) and increased social functioning difficulties (Fett et al., 2011).

Self-monitoring deficits have also been consistently reported along the psychotic continuum of expression (Franck et al., 2001; Brunelin et al., 2007; Lagioia et al., 2011). These studies would be considered to overlap with the cognitive, self-oriented portion of mentalization. Despite these findings, the specific mentalization impairments characterizing psychotic illnesses remain empirically unexplored (Brent et al., 2014a). We will review the developmental evidence suggesting that aberrant mentalization in clinical psychosis is preceded by more subtle alterations during childhood and adolescence, related to distal (schizotypy) and proximal (CHR) manifestations of risk for psychosis. We will further highlight the studies suggesting that those youths with better mentalization skills resolve early sub-clinical psychotic manifestations on their own, possibly through the protective effect of solid developing mentalizing skills.

### Indirect Evidence in Childhood for an Early Association Between Mentalizing Impairments and Vulnerability to Psychosis

Mentalizing deficits have been characterized as trans-diagnostic (Fonagy et al., 2011), yet it remains unclear whether specific mentalizing deficits can be identified along the different stages of psychotic expression. In this regard, early schizotypal expressions such as odd beliefs and ideas of reference may represent the more specific manifestations of early mentalizing aberrations, indicating an increased risk for psychosis. According to Schiffman et al. (2004), the observed premorbid ToM deficits may reflect a general underlying vulnerability for the development of psychopathology (similar to the case of childhood trauma). Another possibility is that intact mentalizing may constitute a resilience factor, protecting against the development of psychotic-spectrum illnesses in individuals who are otherwise at increased risk due to genetic, interpersonal or social influences.

Bartels-Velthuis et al. (2011) examined the moderating role of mentalizing in the development of delusional ideation secondary to abnormal perceptual experiences (AVH) during childhood. They used the ToM Storybook Frank (Blijd-Hoogewys et al., 2008), in which children are tested on their ability to understand first and second-order false beliefs, white lies, irony, deception, double bluffs and faux pas, while being presented with pictures and listening to the story read aloud. Among children experiencing auditory hallucinations at age 7–8 and/or 12–13 years, as measured by the auditory vocal hallucinations scale (AVHRS, Jenner and Van de Willige, 2002), the risk for the development of secondary delusional ideation was significantly higher in those with lower ToM task scores, suggesting that better mentalizing capacities may provide a protective barrier against the development of delusional explanations of unusual perceptual experiences (Bartels-Velthuis et al., 2011).

In another study, Clemmensen et al. (2014) examined ToM using the Danish version of the ToM Storybook Frank, in a sample of 1630 children (11–12 years old) from the general population (study I) and in a case-control based sample of 259 children aged 12–13 years, half of whom reported AVH at age 7–8 years (study II). It was hypothesized that PE measured by the K-SADS-PL (Kaufman et al., 1997) would be specifically associated with a selective mentalizing pattern of hyper-ToM (over-attribution of intentions). In sample I, children with low ToM scores (below the median) were at an elevated risk of experiencing PE compared to children with higher ToM scores. However, this effect was not found in sample II. Pertinently, children who displayed a hypermentalizing style were significantly more likely to experience PE (sample I OR = 2, sample II OR = 1.6) compared to non-hypermentalizing children, and this effect was particularly pronounced for experiences of paranoid/persecutory ideation. According to Clemmensen et al. (2014), the observed hyper-ToM pattern may be stress-induced as studies have shown that increased cortisol production is linked to a hypermentalizing pattern of social cognition (Smeets et al., 2009). In another targeted study, Clemmensen et al. (2016) examined the specificity of the association between hypermentalizing (measured by the Danish version of the ToM Storybook Frank) and PE compared to other known risk factors (i.e., family illness, gender, bullying, changes in family income, etc.), in a sample of 1630 children (11–12 years) from the general population. Analyses revealed that hypermentalizing along with a concurrent psychiatric diagnosis, involvement in bullying and low family income were all associated with PE. Importantly, however, hypermentalizing was the only factor independently associated with PE in the absence of concurrent illness. Moreover, involvement in bullying was associated with an increased risk for PE but was also associated with an elevated risk for the development of general psychopathology without PE. Thus, while most factors associated with PE (like bullying) appear to be linked with a non-specific risk for the development of psychopathology, in this study, hypermentalizing alone appears to be specifically associated with the emergence of psychotic-like experiences (Clemmensen et al., 2016).

These studies are suggestive of a relationship, quite early in development, between mentalization and the first sub-clinical signs of psychosis. Studies involving older adolescents and young adults, which we will review below, further strengthen the case of a developmental interaction between mentalization and risk for psychosis.

### Mentalization During Adolescence and Psychosis High-Risk States

To our knowledge, very little research has focused on metacognitive reasoning about the self in adolescents along the continuum of psychotic expression. Our group has carried out two investigations examining the relationship between self-monitoring difficulties and schizotypy during adolescence (Debbané et al., 2008, 2009). In both studies, we observed a significant association between increased self-reported schizotypy and self-monitoring impairments, suggesting that metacognitive difficulties related to the self may underpin the early expression of risk for psychosis in adolescence. We also found that self-reported schizotypy during adolescence correlated with atypical brain activation patterns in the medial and lateral prefrontal cortices during reality monitoring and self-other trait adjective attribution tasks (Lagioia et al., 2011; Debbané et al., 2014). In studies with older adolescents and adults, self-monitoring deficits have also been identified in the prodromal phase of psychosis (Johns et al., 2001, 2010), indicating that self-monitoring impairments precede the full expression of psychotic psychopathology.

The associations between mentalizing deficits and positive psychotic manifestations in adolescence reported by Barragan et al. (2011) are in line with studies of adult psychometric risk samples. Langdon and Coltheart (1999) found selective ToM deficits among high schizotypal (on the basis of SPQ scores) non-clinical individuals, which were independent of executive planning or inhibitory control deficits. Additionally, Pickup (2006) found that higher positive schizotypy scores predicted subtle ToM deficits in a healthy population sample. No significant associations were found between ToM scores and total or negative schizotypy, suggesting that the relationship between ToM and negative psychotic manifestations may only emerge following the transition to psychosis (Pickup, 2006).

A relatively small amount of research has examined the role of ToM deficits in the transition from the psychosis risk state to the psychiatric form of the illness. Evidence suggests that deficits in social cognition are present in the prodromal phase of psychosis (Thompson et al., 2012) and are often similar to those found among formal psychosis samples (Thompson et al., 2011). Meta-analytic data have shown that similarly to unaffected relatives of patients with psychosis, CHR individuals have significantly impaired ToM compared to healthy controls, but significantly better ToM compared to first episode sufferers (Bora and Pantelis, 2013). Chung et al. (2008) reported significantly worse ToM performance (measured by the False Belief, the Strange Story and a Cartoon tasks) in a CHR sample of young adults (mean age = 20.88) compared to an age and IQ matched healthy control sample, with intermediate effect size levels (0.64–0.68). These findings suggest that ToM deficits may constitute important vulnerability factors underpinning the development of psychosis and highlight the need for prospective studies examining the specific effects of mentalization on the developmental unfolding of psychotic disorders.

In a longitudinal study investigating ToM in a group of 49 subjects at CHR for psychosis, Kim et al. (2011) observed that CHR individuals who transitioned to psychosis over a 5.2 year-period displayed worse baseline scores on False Belief, Strange Story and cartoon ToM tasks, as well as on a number of neurocognitive assessments, compared with non-converters. Moreover, the authors reported that a model combining both ToM and neurocognitive scores significantly predicted the time of transition to formal psychosis. Contrary to the data reported by Kim et al. (2011), recent longitudinal evidence in CHR from the large NAPLS-2 cohort (Piskulic et al., 2016) indicates that differences in social cognition alone, may not be sufficient to predict the transition from prodromal psychotic manifestations to the clinical form of the illness. Although the CHR group (*n* = 764) as a whole displayed significantly worse performance in various domains of social cognition (ToM, social perception, facial emotion perception) compared to healthy controls (*n* = 280), no significant group differences emerged in any of the social cognitive domains between CHR individuals who transitioned to psychosis and their non-converting counterparts (Piskulic et al., 2016). Importantly, however, improvements in social cognition over time (one year follow-up) were more prominent in CHR non-converters and healthy controls compared to CHR converters.

These prospective longitudinal studies are essential to further our understanding of early social cognitive development that precedes the emergence of psychotic disorders. It appears that impairments in mentalization are not consistent “predictors” of transition, but may signal a breakdown in resilience factors protecting against the emergence of psychosis. Still, many of the studies reviewed in this section focus on state manifestations of psychosis risk (positive psychotic-like symptoms), which are not as enduring as trait risk factors represented by endophenotypes (such as sensory gating abnormalities, neurological soft signs, social and physical anhedonia) that are evidenced in individuals at genetic risk for psychosis (Gottesman and Gould, 2003). According to a recent model (Debbané et al., 2015), state-risk manifestations may reflect a series of developmental interactions or exacerbation of an underlying schizotypal trait-liability (Rado, 1953; Meehl, 1962) that is dimensionally distributed in the general population at different degrees (from complete absence and schizotypal personality characteristics to the extreme of clinical psychotic symptomatology). It is thus conceivable that sub-optimal mentalization capacities, often found in the context of insecure attachment, may combine with the underlying schizotypal “seed” or liability, to undermine resilience processes protecting against the transition to clinical psychosis. In this way the degree of psychosis expression and the derailment of social-cognitive processes would be conceptualized as a developmental transaction, converging towards a clinical psychosis outcome. Below, we further consider how computational psychiatry may contribute in bridging the divide between cognitive impairments and sensory-based endophenotypes along the trajectory of risk for psychosis.

### A Computational Framework for Integrating Attachment, Neurobiology and Mentalization

In this final section, we will propose that insecure attachment may be connected to disturbed and potentially psychotic experience of oneself through mechanisms subsumed under the umbrella term of *embodied mentalizing*, an expression that translates the processes needed to *detect*, *identify* and *regulate* signals coming from one’s body to harness them with one’s mind. The concept of embodied mentalizing translates early insights from the field of psychoanalytic psychosomatics suggesting that mentalization represents a suite of processes responsible for the regulation and transformation of physiological activation through higher-order psychological processes (Marty, 1991; Lecours and Bouchard, 1997). Recent conceptualizations have emphasized the role played by embodied mentalizing in the development and treatment of functional somatic disorders (Luyten et al., 2012; Luyten and Fonagy, 2016) and depression (Luyten et al., 2013). We argue that the concept of embodied mentalizing may help bridge the gap to understand how early signs of risk for psychosis may link to later emerging psychotic states during the CHR and psychosis onset, and most importantly, to severely impaired social cognition.

As in high-risk psychosis research, studies in clinical developmental neuroscience distinguish two levels of psychotic impairment that may be usefully articulated in the context the embodied mentalizing hypothesis. First, at the “trait” level of liability to psychosis, processes of neuromotor integration (“neurological soft signs”; Compton et al., 2007) and sensory gating, represent well-known endophenotypes of psychosis, which are observable in first-degree relatives and along the continuum of severity in psychotic disorders (Braff et al., 2001). These endophenotypes are linked to both genetic vulnerability as well as to developmental oxidative stress for which, as reviewed above, attachment adversity may act as one factor promoting early impairment. At the second level, sub-clinical to clinical “state” symptoms, encompassing subclinical psychotic-like phenomena, CHR manifestations, and frank hallucinatory or delusional states, may be best characterized as false inferences emerging from a failure to regulate the relationship between certainty about *prior belief* and certainty about sensory experience (Adams et al., 2013). We would like to underline here that we will employ the expression “*sensory-affective*” experiences to account for the lived experience pertaining to fear, hostility and anguish underpinning PE; importantly, sensory-affective experiences permeating psychotic manifestations are often triggered in the context of intimate relationships, such as closeness, attraction, and sexual arousal which are most often difficult to tolerate and subject to avoidance in people suffering from psychosis, because these experiences typically carry the disorganizing effect of blurring self-other boundaries (Fonagy, 2008).

Sensory-affective experience is key to a contemporary, developmental, and psychodynamically-oriented understanding of the emergence of psychosis. In this framework, mentalization constitutes an interesting construct by virtue of the fact that it seeks to account for the dynamic connectivity between systems sustaining low-level embodied experience and systems sustaining superior cognitive inferential processes, which constantly interact to shape one’s understanding of self and others’ behaviors. As we will argue on the basis of contemporary neuroscience (Palaniyappan et al., 2012; Seth, 2013), different forms of **imbalance** between certainty about sensory-affective experience and certainty about prior beliefs in oneself may help explain aberrant *embodied mentalizing* along the psychosis continuum, where too little or too much weight to either prior beliefs or sensory-affective evidence could sustain both trait and state expression of psychosis from sub-clinical to clinical manifestations.

Another important issue from our perspective relates to psychosexual development from adolescence to adulthood, in parallel to the neural maturation and specialization of cerebral networks. On the basis of clinical conceptualizations (Fonagy and Luyten, 2016), we suggest that among individuals at-risk for psychosis and in the context of early attachment adversity, difficulties in embodied mentalizing may become particularly relevant during adolescent development, which is characterized by the initiation of intimacy in interpersonal relationships, together with psychophysiological changes which profoundly modify the youth’s position in two domains: sexuality and aggression (Laufer and Laufer, 1995). Whereas before puberty the domains of sexuality and aggression were less integrated in the agentive sphere of the individual, their potentiation through pubertal development gradually brings novel possibilities: procreation and potentially causing enduring harm through one’s aggression. This poses an inherent challenge for mentalizing during adolescence, as body-states associated with new experiences of sexual and aggressive arousal remain without fully regulatory second-order representations, unlike other forms of affective arousal stemming from basic emotions which have been mirrored throughout childhood and internalized in the self-regulatory repertoire (Target, 2007; Fonagy, 2008; Fonagy and Allison, 2016; Fonagy and Luyten, 2016). This makes new arousal states in adolescence prone to poorly regulated thought and behavior, especially in situations of stress (Fonagy and Luyten, 2016). Under favorable circumstances, adolescents with relatively stable mentalizing capacities, manage to utilize the widening of interpersonal relationships (peer, romantic) during adolescence, to further elaborate their ability to form representations of their own and other people’s mental states, and to integrate their sexual and aggressive states in their developing identities.

Importantly however, children who have experienced sexual or physical abuse in the context of early attachment relationships, enter adolescence with a severely undermined mentalizing capacity (Cicchetti et al., 2003; Ensink et al., 2015). For these youths, adolescent-specific confrontations with unmentalized feelings of aggression and sexuality, and more importantly, with the bodily-states that accompany them, may produce severe distress, leading to further disruptions of mentalizing and the re-emergence of prementalizing modes of psychic reality as a means of coping with painful interpersonal experiences.

In order to develop the hypothesis that embodied mentalizing constitutes an important moderator of the relationship between attachment adversity and psychosis, we will further specify the computational psychiatric framework of psychosis as critically involving the relationship between certainty of the sensory (sensory-affective states) and belief (cognitive states) systems, and conclude by presenting a heuristic model of embodied mentalizing as a moderator of psychotic manifestations along their continuum of severity.

### A Computational Framework for the Psychosis Continuum

The clinical developmental course of psychotic disorders, illustrated earlier in Figure 1, delineates periods characterized by sub-clinical and clinical manifestations associated with risk and onset of psychosis. At a descriptive level, these manifestations can be differentiated into relatively stable, *trait* abnormalities (such as the psychophysiological endophenotypes evidenced in both people with schizophrenia and their non-affected family members), and more transient state manifestations (such as thought disturbances and cognitive-perceptual abnormalities). In linking the descriptive and developmental perspectives, two important observations may be underlined. First, trait abnormalities involve a range of subtle impairments acting upon normative psychophysiological, sensorimotor and basic affective drive functions which presumably all reach maturity before puberty (Debbané and Barrantes-Vidal, 2015). For example, the schizophrenia endophenotype known as prepulse inhihibition characterizes abnormal sensory gating function, and is thought to reach adult-like levels around 8 years of age (Braff et al., 2001). Endophenotypes typically do not correlate with state manifestations of psychosis (Gottesman and Gould, 2003); they more likely influence the developmental cascade of higher order cognition, such as metacognition or mentalization, whose failures are apparent in the cardinal symptoms of psychosis. Second, these endophenotypes typically act as stable procedural regulation mechanisms for low-level processing. This would suggest that children at high-risk for psychosis (i.e., CHR or genetic high-risk individuals) develop higher cognitive functions (executive and social cognition) in the context of these subtle dysfunctions. It further suggests that at-risk individuals may need to compensate for slight abnormalities in the earliest stages of information processing, through higher-order cognition such as mentalization, appraisal, and/or metacognitive mechanisms (Debbané et al., 2016).

From a computational perspective, trait abnormalities along the continuum of psychosis have been framed as the brain’s impairment in consistently predicting sensory input (Adams et al., 2013). With regards to sensory gating impairments, larger P50 responses confront the psychosis-prone individual with experiences of “surprise” in the context of closely contingent stimuli presentations. Later, along the information processing trajectory, reduced discriminatory responses evidenced by mismatch negativity or oddball paradigms speak to increased difficulty in discarding irrelevant information. Critically, such subtle impairments in early processing may significantly challenge higher cognitive functioning downstream in the information processing pathway. Following this hypothetical proposition, Kantrowitz et al. (2014) provide an empirical illustration of how early sensory processing impairments in patients with schizophrenia-spectrum disorders disturb higher order cognition, using the case of perception of sarcasm (Kantrowitz et al., 2014). The authors employ an experimental auditory functional neuroimaging task to measure sarcasm processing, in relation to two important sub-processing, namely pitch processing and emotion recognition. They find that early auditory processing impairments significantly contribute to decreased sarcasm perception in the patient group, independent of general cognitive impairment. Functional connectivity within the patient group’s auditory processing network, but not within the core mentalizing network, correlated significantly with impaired sarcasm perception. This study nicely illustrates how subtle impairments in early sensory processing may, alter downstream, the deployment of accurate mentalizing. In control participants, functional connectivity between mentalizing core regions, including areas dedicated to embodied mentalizing (i.e., insular cortices) were engaged in sarcasm perception, suggesting the importance of assessing the cross-talk between the sensory-affective and cognitive processing regions when examining accurate mentalizing.

At the computational level, the relationship between the sensory and cognitive signals can be studied using a normative (Bayes-optimal) account of cerebral functioning. Adams et al. (2013) propose a computational account of the anatomy of psychosis, articulated within a hierarchical predictive coding model that may contribute, in part, to further examine the nature of trait and state manifestations of psychosis (Adams et al., 2013). Predictive coding assumes that the brain is an inference machine seeking to reduce its prediction error (Friston, 2010). From this perspective, the brain encodes a number of *priors* (e.g., prior beliefs, mentalizing working models about self and other, and/or metacognitive beliefs) on the basis of experience, and hierarchically construes a virtual interpretative algorithm, which continues to refine itself when it comes in contact with information that signals significant error of prediction. In technical terms, refinement of priors augment their *precision*, and putatively, the experience of certainty. Sensory-affective perception is characteristically precise and interpreted as “real” with an important degree of certainty, because it is easily accessible and can be reliably predicted. In our general experience, the brain encodes that our sensory experience is real (our senses do not lie), their signals are highly reliable. However, prior beliefs attenuate the experience of sensory precision, as for example, our cultural belief that the senses should not be fully trusted, emanating from Cartesian philosophy. The prediction algorithm’s goal is to minimize surprise (error), and pursue its hierarchical elaboration to accurately compute increasing complexity that may be used to navigate within the world and interpret new information accurately.

**Figure 3:**
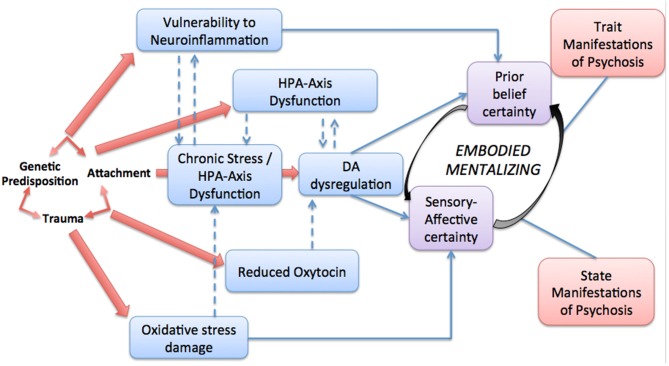
**Illustration adapting Figure 2 to account for the interaction between sensory and beliefs priors influencing each other’s precision (certainty); at different levels of analysis, this relationship is foundational to embodied mentalizing, and may differentially sustain early trait vulnerability, and later state manifestations of psychosis**.

In this account, (Figure 3) trait manifestations of psychosis risk, represented by endophenotypes, are conceptualized as the result of failures of top-down priors to attenuate the sensory-affective precision. As mentioned earlier, basic psychophysiological function such as sensory gating regulate the novelty or oddness of predictable (most often sensory) information. In computational terms, basic sensory gating mechanisms regulate the precision (certainty) of the sensory signal. In cases of subtle failures of these low-level regulatory mechanisms, sensory experience is attributed more precision (increased surprise, stronger certainty) and requires a top-down mechanism to attenuate certainty. In cases of weak prior beliefs (in other words, in cases of sub-optimal mentalization and/or metacognitive function—such as in children, or in at-risk individuals), even predictable sensory signals may be experienced as somewhat newer, more surprising, and perhaps with a stronger sense of realness. Typical development of metacognitive skills, including mentalization, diminish the propensity to experience self-generated content as surprising or magical (Bartels-Velthuis et al., 2011; Debbané et al., 2013). The role of embodied mentalizing in regards to trait manifestations of psychosis would be to attenuate the sensory precision emanating from subtle impairments in low-level sensory gating regulation.

Turning now to CHR state symptoms, such as subclinical hallucinations, the computational framework argues that they may result from an increase in prior belief precision, such that they remain impervious to contrary (imprecise) sensory-affective evidence (Adams et al., 2013). Again, the focus is on the relationship of priors relative to sensory-affective precision that is key. Note that hallucinations could also result from a failure of the corollary discharge to attenuate the sensory precision of self-generated acts, such as inner speech (Fletcher and Frith, 2009). The case of delusions may be compensatory in nature, as a way for the system to discard overwhelming “surprise”, i.e., sensory-affective precision of disturbing nature. Delusional explanations secondary to perceptual aberrations could fall under this explicative model. In essence, state symptoms of psychosis more directly involve the top-down component, which recruits a form of dis-embodied mentalizing where the sensory-affective signal is discarded to regulate its disturbing effects through extreme certainty in prior beliefs. This contemporary account resonates with the concept of “splitting” as a psychological defense mechanism put forward by psychoanalysis to account for psychotic functioning (Freud, 1911; Klein, 1946; Bion, 1957). The computational approach to splitting may enable direct and fine-grain hypothesis testing of different psychotic phenomena.

To summarize, the concept of embodied mentalizing provides a bridge to the theoretical propositions put forward by computational psychiatry, as it represents the integration of sensory-affective and metacognitive signals to modulate certainty of each, ensuing updating of beliefs and guidance of behavior. Further research is required to determine the mechanisms by which the interplay between the sensory-affective and metacognitive signals occur at different levels of analysis (cellular, network, systems and psychological levels).

## Conclusion

This article provides a comprehensive review of the literature linking attachment, neurobiology and mentalizing along the continuum of psychosis expression, from the premorbid subclinical manifestations to full-blown psychotic disorder. This review has led us to propose an integrative model of psychosis based on three key assumptions: (1) attachment security constitutes a non-specific protective factor in individuals at increased risk for psychosis.; (2) Disturbed attachment can impact on at least five different neurobiological pathways implicated in sustaining self and other mentalizing; and (3) Embodied mentalizing may serve as a moderating factor in the expression of psychosis.

At the interpersonal level, attachment security is associated with help-seeking behavior and with more favorable outcomes in individuals suffering from psychosis. In the preclinical stages of individuals at risk for psychosis, attachment security is associated with less severe manifestations of early trait signs (schizotypy). At the psychological level, attachment security provides a key developmental context to acquire the building blocks for robust social cognition and mentalizing. Disruptions in attachment are often linked to early trauma as well as bullying during adolescence. In some individuals, developmental adversity may promote the use of anxious, avoidant, or even disorganized attachment strategies. While these strategies may constitute adaptation attempts to adverse and hostile environments, they tend to undermine the development of the capacity to attend to one and others’ minds (mentalizing), and they affect the unfolding of social cognitive skills. We suggest that the oft-cited relationship between insecure attachment, trauma and psychosis does not represent a causal, etiological chain towards psychotic disorders, but rather, a transactional process taking place during development, which increases the risk of transitioning to psychosis in at-risk individuals, by virtue of detrimental effects on self and other mentalizing during childhood and adolescence, particularly in relation to embodied mentalizing.

Second, the proposed integrative model suggests five developmental pathways through which disturbed attachment may affect the neurological integrity of key networks sustaining self and other mentalizing in individuals at risk for psychosis. Specifically, impairments in the HPA-axis, dopamine dysfunction, reduced oxytocin levels, neuroinflamation and oxidative stress all may constitute potential pathways through which genetic, interpersonal and environmental risk may impinge on otherwise vulnerable individuals. At the same time, adversity in early attachment has been shown to impact the severity of neuroinflammation, oxidative stress and HPA-axis dysfunction. These impairments may further interact with the dopaminergic and oxytonergic systems, known to be involved in regulating the reward and the salience of relationships in particular. Most importantly perhaps, these five neurobiological pathways are further involved in developmental alterations of sensory-affective processes (basic sensory-gating and affect regulation processes) as well as higher-order cognitive processes (reality monitoring and metacognitive reasoning) underpinning trait and state symptoms of psychosis.

Finally, we have pointed to the potential moderating role of embodied mentalizing in the expression of psychosis. Embodied mentalizing refers to the capacity of experiencing the body as the seat of emotional responses; as an active process it aims to reflectively detect, identify and regulate signals coming from one’s body to harness them with one’s mind. Recent advances in computational psychiatry have revived the interest in the relationship between sensory-affective experience and belief-reasoning (cognitive) processes. Normative functioning involves co-regulation between sensory-affective and cognitive processes to sustain the development of complex cognition, and also to prevent aberrant interpretations of experience. In the case where sensory-affective experience fails to be harnessed by regulatory or compensatory cognitive mechanisms, it can come to dominate subjective experience as expressed in trait symptoms of psychosis (from endophenotypes to negative symptoms). In cases where cognitive priors dominate and fail to be modulated by sensory-affective experience, state manifestations of psychosis are more likely to dominate the experience of the individual (from magical thinking to frank delusional ideation). The co-regulation between sensory-affective and cognitive, which can be subsumed under the notion of embodied mentalizing, may thus constitute a moderating set of processes along the continuum of psychosis expression.

The present model may assist in hypothesis building for neuroscientific research purposes, and also further inform the development of preventive treatment methods that target mentalizing as a protective factor, or psychotherapeutic methods focussing on mechanisms of change (Brent and Fonagy, 2014; Debbané et al., 2016; Weijers et al., 2016). Most importantly perhaps, treatment schemes such as mentalization-based treatment (MBT) may integrate a more pragmatic treatment paradigm in the early phases of an emerging psychosis promoted by the stage-sensitive conceptualization for treatment (McGorry et al., 2007). In its current form, the “clinical staging approach”, which is extensively used for other progressive medical diseases, leaves many questions open, especially with regards to the active ingredients, also known as *mechanisms of change*, at every treatment stage of emerging psychotic disorders. Working from the bottom-up, the staging approach may benefit from therapeutic models that are developmental in nature, in order to achieve the kind of personalized-medicine it seeks to promote (McGorry, 2013). It may be that psycho-therapeutic care targeting mentalizing or social cognition may be superior to the general psychosocial care currently proposed for the early stages corresponding to CHR for psychosis. The latter remains an empirical question that will have to be examined as we enter a new stage of developing preventive treatments for psychosis.

## Author Contributions

All authors contributed to the conceptualization of the review. Each author worked specifically on a sub-section of the review, contributing literature review and writing. MD as well as GS merged the sub-sections and wrote the draft. All authors contributed in refining the final draft and approved of its contents.

## Funding

For this work, MD was supported by the Swiss National Science Foundation (100019_159440) and Gertrude Von Meissner Foundation (ME 7871).

## Conflict of Interest Statement

The authors declare that the research was conducted in the absence of any commercial or financial relationships that could be construed as a potential conflict of interest.
